# N‐Aromatic Complexation in Tetraphenyl Porphyrin Iron (III)‐Pyridine: Evidence of Spin‐Flip via Gas‐Phase Electronic Spectroscopy

**DOI:** 10.1002/cphc.202400669

**Published:** 2024-11-08

**Authors:** Kelechi O. Uleanya, Sarah A. Wilson, Caroline E. H. Dessent

**Affiliations:** ^1^ Department of Chemistry University of York Heslington, York YO10 5DD United Kingdom

**Keywords:** Metalloporphyrin, Molecular orbitals, Excited states, Density functional theory, Mass spectrometry

## Abstract

There is growing interest in the electronic properties of metalloporphyrins especially in relation to their interactions with other molecular species in their local environment. Here, UV‐VIS laser photodissociation spectroscopy in vacuo has been applied to an iron‐centred metalloporphyrin (FeTPP^+^) and its *N*‐aromatic adduct with pyridine (py) to determine the electronic effect of complexation. Both the metalloporphyrin (FeTPP^+^) and pyridine adduct (FeTPP^+^⋅py) absorb strongly across the spectral region studied (652–302 nm: 1.91–4.10 eV). Notably, a large blue shift was observed for the dominant Soret band (41 nm) upon complexation (0.47±0.02 eV), indicative of strong pyridine binding. Significant differences in the profiles (*i. e*. number and position of bands) of the electronic spectra are evident comparing FeTPP^+^ and FeTPP^+^⋅py. Time‐dependent density functional theory calculations were used to assign the spectra, revealing that the Fe^III^ spin‐state flips from S=3/2 to S=5/2 upon complexation with pyridine. For FeTPP^+^, all bright spectral transitions are found to be π‐π* in character, with electron density variously distributed across the porphyrin and/or its phenyl substituents. Similar electronic excitations are observed for FeTPP^+^⋅py, with an additional bright transition which involves charge transfer from the porphyrin to the pyridine moiety.

## Graphical abstract

Laser photodissociation spectroscopy in vacuo has been applied to an iron‐centred metalloporphyrin (Tetraphenyl Iron (III) Porphyrin, FeTPP^+^) and its *N*‐aromatic adduct with pyridine to determine the electronic effect of complexation, revealing that the Fe^III^ spin‐state.flips from S=3/2 to a higher‐spin S=5/2 state upon pyridine binding.

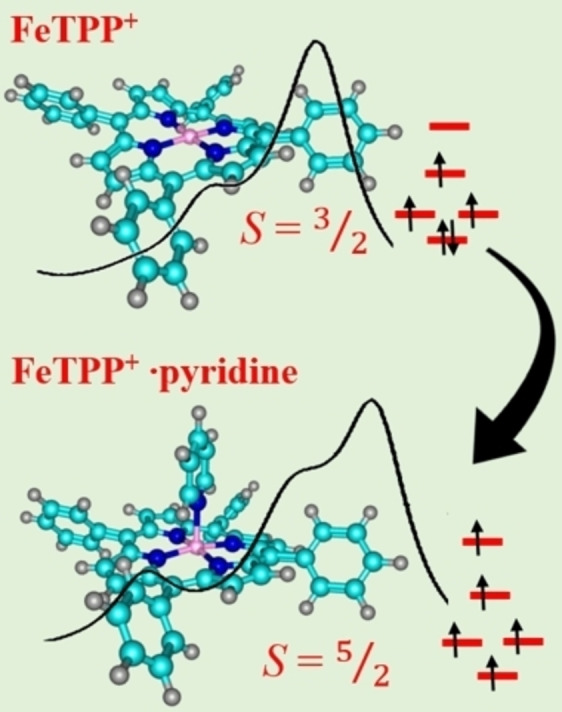

## 1 Introduction

Metalloporphyrins (MPs) constitute an important class of bio‐inorganic compounds that are under intense current investigation for applications ranging from photomedicine to optoelectronic materials.[^1^, ^2^, ^3^, ^4^, ^5^, ^6^, ^7^, ^8^] While there is strong interest in their photophysical properties,[^7^, ^8^] MPs exhibit rather narrow absorption bands across the UV‐VIS spectral range. There is therefore considerable interest in modifying MP structures in a controlled fashion so that the position and width of the porphyrin absorption bands can be tuned with interest in impact of the local environment on electronic properties.[^7^, ^8^, ^9^, ^10^, ^11^] A fundamental understanding of the nature of MP excited states is essential to inform the rational design of new metalloporphyrin materials.

The vast majority of studies of the UV‐VIS spectroscopy of MPs conducted to date have been performed in the solution‐phase, with studies providing information on general trends in the positions and intensities of the electronic bands as a function of porphyrin derivatization.^[12]^ However, there are numerous factors that can hamper the interpretation of a solution‐phase spectrum, including direct solvent coordination to the porphyrin metal centre, counterion interactions with the MP and also aggregation of the porphyrins.[^13^, ^14^, ^15^] In such situations, gas‐phase experiments on isolated molecular species can provide unrivalled, unambiguous information on the intrinsic electronic spectrum in the absence of bulk‐solution complications.[^16^, ^17^] Indeed, a gas‐phase experiment is ideal for establishing the fundamental structural factors that influence an electronic spectrum. The effect of solvent (or other coordinating ligands) on the spectrum can then be reintroduced in a step‐wise fashion through the study of microsolvated molecular clusters.[^18^, ^19^]

The field of petroleum science provides an interesting illustration of the issues that can surround interpretation of the electronic spectra of MPs. Metal‐containing porphyrins are the dominant inorganic components of fossil fuels (10^3^–10^4^ ppm),[^20^, ^21^, ^22^, ^23^] and are of key fundamental interest as robust, inert biomarkers, providing geochemical information on the deposition environment.^[23]^ However, the detection of MPs via UV‐VIS spectroscopy in crude oil has proven difficult, since although MPs can in principle be readily identified by their distinctive “Soret” band absorption in the near‐UV, these bands are frequently much weaker and broader in fuel samples than would be expected from the known concentrations.[^24^, ^25^] Several hypotheses have been put forward to explain this. Dickie et al. proposed that MPs stack with aromatic sheets of polycyclic asphaltene compounds, thus reducing the observed excitation coefficients.^[25]^ Stoyanov et al. subsequently proposed that the Soret band may be modified due to attachment of larger aromatics and other asphaltene molecules, either through axial coordination of the metal center or fusion (annelation) to aromatic rings on the porphyrin π‐system.[^26^, ^27^]

**Scheme 1:**
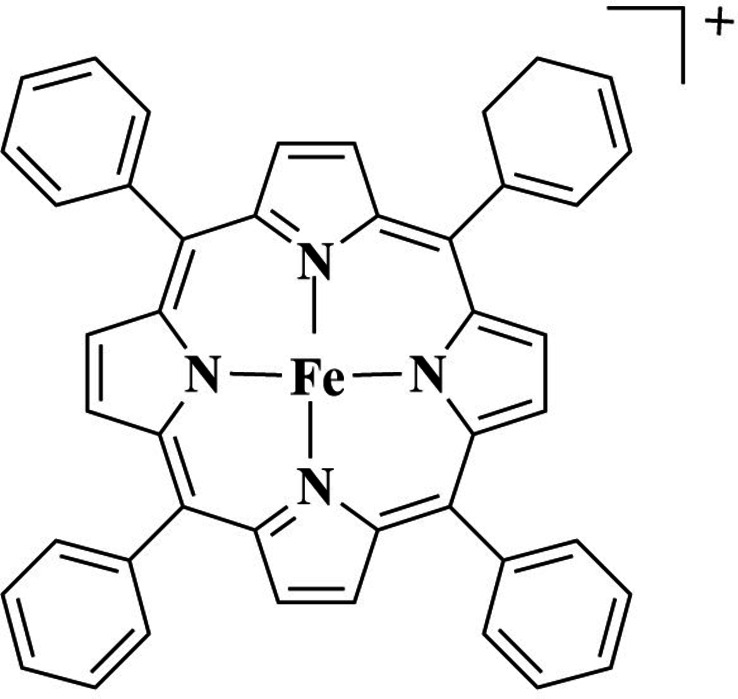
Schematic diagram of the tetraphenyl porphyrin Fe (III) cation (FeTPP^+^).

In this work, we use gas‐phase laser photodissociation spectroscopy to test how axial binding can affect the nature of the MP electronic transitions directly by acquiring the UV‐VIS photodepletion spectrum of Tetraphenyl Iron (III) Porphyrin (FeTPP^+^) and comparing it to that of the FeTPP^+^⋅pyridine adduct. This allows us to assess the extent to which N‐aromatic complexation affects the strength of the MP electronic transitions over a broad spectral range from 302–652 nm (1.91–4.10 eV), as an unambiguous test case of how MP absorption can be affected by aromatic binding. Scheme 1 shows the chemical structure of the metalloporphyrin studied in this work. Pyridine is selected as the aromatic molecule since it is a known component of petrochemicals,^[28]^ and has the potential to coordinate *via* an axial interaction to the MP metal centre. In addition to the immediate question of how axial aromatic complexation affects the electronic properties of an MP, adducts of iron metalloporphyrins are also of interest in biochemistry as models of anti‐malarial drug action.^[29]^

A limited number of MP‐molecule adducts have been studied spectroscopically, in vacuo previously, via either UV‐VIS, IR or photoelectron spectroscopy. However, these studies have all involved smaller molecules as ligands, either simple solvent molecules (e. g. water, methanol, acetonitrile) or small inorganic ligands (e. g. NO, CO, O_2_).[^30^, ^31^, ^32^, ^33^, ^34^, ^35^] One of the key results to emerge from these studies has been the extent to which electronic bands undergo spectral shift upon complexation to small molecules.[^30^, ^31^, ^32^] We also note that Dugourd and co‐workers have studied intact cytochrome c protein anions in vacuo (containing a heme MP), and found that the gas‐phase absorption of the heme porphyrin chromophore is similar to that of the native protein in solution.^[36]^ Shafizadeh and co‐workers have conducted several gas‐phase studies to measure binding energies of ligands to iron metalloporphyrins within cooled ion traps.[^37^, ^38^, ^39^, ^40^] This work is reviewed in Ref,^[40]^ and has revealed that the CO and O_2_ ligands are weak‐binding ligands, with H_2_O, NO and imidazole representing medium to strong binding ligands. The work presented here is the first to incorporate extensive time‐dependent density functional theory (TDDFT) calculations of the MP and its’ adduct to provide a deeper insight into the nature of the electronic transitions.

## 2 Experimental and Computational Methods

Experiments were conducted using a modified Bruker AmaZon quadrupole ion trap and Orbitrap Fusion Tribrid mass spectrometer, as described in detail previously.[^41^, ^42^, ^43^] FeTPP^+^⋅py clusters were prepared by electrospraying solutions of 1×10^−6^ M FeTPPCl and 20 μL of the relevant 1×10^−3^ M pyridine in acetonitrile (MeCN). All chemicals used were purchased from Sigma Aldrich and used without further purification. Solution‐phase UV absorption spectrum of FeTPP^+^ 1×10^−6^ mol dm^−3^ in acetonitrile (MeCN) was acquired using a UV‐1800 UV‐vis spectrophotometer (Shimadzu, Kyoto, Japan) with a 1 cm cuvette. Higher‐energy collisional dissociation (HCD) was conducted in the Orbitrap,^[43]^ using the automatic tuning capabilities of the operating software at a flow rate of 5 μL min^−1^ in positive ion mode; spray voltage, 3500 V; sweep gas flow rate, 1 arb; sheath gas flow rate, 10 arb; auxiliary gas flow rate, 5 arb; ion transfer tube temperature, 250 °C; vaporiser temperature, 300 °C.

UV‐VIS photons were produced by an Nd:YAG (10 Hz, Surelite) pumped OPO (Horizon) laser, giving 0.05–0.5 mJ of laser energy across the range 3.86–1.95 eV (320–636 nm). Scans were conducted using a 2 nm step size with photofragmentation experiments done at ion accumulation time of 10 ms and a fragmentation time of 100 ms, ensuring an average of one laser pulse per ion packet.[^41^, ^42^] A 0.6 neutral density filter (NDF) was used to reduce the light intensity entering the mass spectrometer ion‐trap resulting in fractional transmittance of 25 %, to minimise the possibility of multiphoton absorption. This was tested by conducting laser‐energy measurements to ensure whether there was a linear relationship between photodepletion intensity and photon energy (Section S1), consistent with single‐photon excitation.

Photodepletion (PD) ion intensity and photofragmentation (PF) production ion intensity were acquired simultaneously and were calculated using Equations (1a and 1b,[^41^, ^42^]

(1a) $$Photodepletion\;Intensity=\;\frac{ln\left(\frac{I_{OFF}}{I_{ON}}\right)}{P\lambda }$$

(1b) $$Photofragment\;Production=\;\frac{ln\left(\frac{I_{FRAG}}{I_{OFF}}\right)}{P\lambda }$$

where *I*_OFF_ and *I*_ON_ are the peak intensities with the laser off and on respectively, *I*_FRAG_ is the photofragment intensity due to laser irradiation, *λ* is the excitation wavelength (nm) and P is the laser pulse energy (mJ). All PD and PF intensities reported are an average of three repeat. The % photodepletion intensity and % photofragment yield were calculated using Equations (2a and 2b) respectively.^[44]^

(2a)

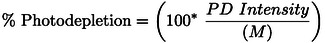




where M is the 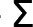 PD intensities at different photon energy
(2b)

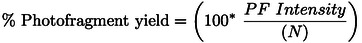




where N is the 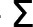 Pf intensities of different fragments at same photon energy

Initial calculations were performed to determine the geometric structures and relative energies of FeTPP^+^ and FeTPP^+^⋅py at the M06‐2X/6‐31G (d,p) level of theory in Gaussian 09,^[45]^ with Fe^III^ in the S=1/2 (low), S=3/2 (intermediate) and S=5/2 (high) spin‐states. The M06‐2X functional was chosen for calculating the geometric structures since it is known to perform well for systems with ionic and dispersion intermolecular interactions,^[46]^ while the modest‐size basis set was selected to balance the computational cost. Frequency calculations were performed to ensure that the optimised structures correspond to true minima. Excited‐state calculations (TDDFT) were performed to calculate vertical excitation energies and simulate absorption spectra, and hence gain insight into the variation of the electronic spectra as a function of electronic spin state. These calculations (70 states, singlets) were performed at the PBE0/6‐31G(p,d) level of theory using QChem.^[47]^ PBE0 was selected for the TDDFT calculations as it is known to perform well for predicting electronic spectra.[^48^, ^49^]

## 3 Results and Discussion

### 3.1 Density Functional Theory Calculations

Table 1 displays the lowest‐energy calculated geometric structures for FeTPP^+^ and FeTPP^+^⋅py with the Fe^III^ spin states of S=1/2, 3/2 and 5/2, along with their relative energies. The calculations predict that S=3/2 is the lowest‐energy state for FeTPP^+^,^[50]^ with S=1/2 and S=5/2 lying somewhat higher in energy, but with energies that are relatively close together. This result agrees with previous calculations on similar systems,[^37^, ^49^] with condensed‐phase experiments also indicating that the 3/2 state represents the ground electronic state.[^39^, ^40^, ^51^, ^52^] The calculations predict a reordering of energies for the three spin states upon complexation with pyridine, with the low‐spin S=1/2 state now being predicted to be the electronic ground state. It should be noted that there are frequently disagreements between calculations and experiment in relation to the relative energetic ordering of the spin states for such systems.[^39^, ^50^, ^51^, ^52^, ^53^, ^54^] Indeed, metal‐centred porphyrins remain a significant challenge for computational chemistry in terms of predictions of spin‐state energies, although other features (e. g. geometric parameters, TDDFT predicted electronic spectra) can be calculated with good agreement to experiment.^[54]^

**Table 1:** Lowest‐energy geometric structures of FeTPP^+^ and FeTPP^+^⋅py displayed with their relative energies (kJ/mol) calculated at the M06‐2X/6‐31G (d,p) level of theory for the Fe^III^ S=1/2, 3/2 and 5/2 spin states.^[a,b]^

Spin States	S=1/2	S=3/2	S=5/2
**FeTPP^+^**	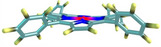 **E=131.7**	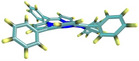 **E=0**	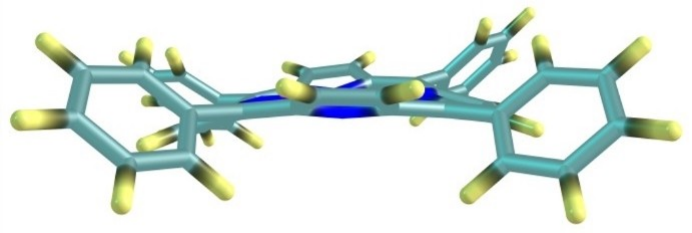 **E=133.5**
**FeTPP^+^⋅py**	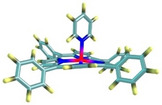 **E=0** **R=1.96 Å**	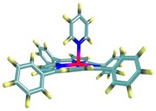 **E=168.0** **R=2.12 Å**	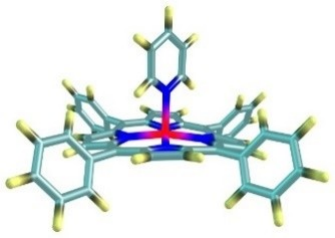 **E=394.4** **R=2.15 Å**

[a] Relative energies are zero point corrected. [b] R=Fe−N bond length.

The iron atom of FeTPP^+^ is centrally located in the porphyrin ring in all structures (Table 1), with the tetrapyrrole ring distorted from a planar geometry.^[39]^ This distortion is due to the metal ion size relative to the strain‐free distances between the central nitrogen atoms, as well as due to steric effects that arise from the phenyl substituents. These structures display different degrees of above‐the‐plane and below‐the‐plane distortion of the phenyl substituent groups in different spin‐state structures, consistent with previous results.^[39]^ For the calculated lowest‐energy structure for FeTPP^+^, S=3/2, there is an out‐of‐plane distortion of the four pyrrole rings of the porphyrin moiety and the highest degree of above‐the‐plane and below‐the‐plane distortion of opposite (i. e. across the porphyrin ring) phenyl groups, respectively. (Tables S1 and S2 provide additional views of the structures.)

For the FeTPP^+^⋅py adducts (Table 1), the lowest‐energy geometric structure displays the expected axial *N*‐coordination of the aromatic to the Fe‐centre of the MP with the Fe atom displaced out‐of‐plane with respect to the porphyrin ring. Notably, the longest Fe−N distance is observed for the 5/2 structure, consistent with the expected electron repulsion that will be present in the high‐spin state.^[53]^ The Fe−N values are within the range obtained computationally for *N*‐donor binding to another Fe^III^ porphine with axial co‐ordination by Durrant,^[29]^ and Shafizadeh and co‐workers.[^39^, ^40^] It is notable that the phenyl substituent groups are less distorted from the plane of the porphyrin ring in the adducts compared to the metalloporphyrin.

There is a distinct difference between the position of the Fe atom in the MP compared with the adduct for all spin states of FeTPP^+^⋅py, with the Fe atom displaced further out of the plane of the four porphine ring N atoms upon pyridine coordination. The same out‐of‐plane displacement of the Fe atom was observed for the related Fe^III^ protoporphyrin‐pyridine adduct studied by Durrant previously.^[29]^ The interaction strength of the Fe atom with both the porphyrin ring and the axial ligand in an adduct is dependent on the nature of the axial ligand and the substituents attached to the porphyrin. Indeed, it has been proposed that the withdrawal of π‐electron density from the porphyrin ring strengthens iron to porphyrin π‐bonding and thereby weakens the π‐bonds of the iron to axial ligands and vice versa.[^38^, ^55^] Since pyridine has a lone pair of electron to donate, this can result in stronger iron to axial ligand π‐bonding and thus a weaker iron to porphyrin π‐bonding. This type of interaction likely drives the reduction in distortion of the phenyl substituent groups that is observed for the adduct compared to FeTTP^+^ that is evident in the structures presented in Tables S1 and S2 (Sections S2 and S3).

**Figure 1:**
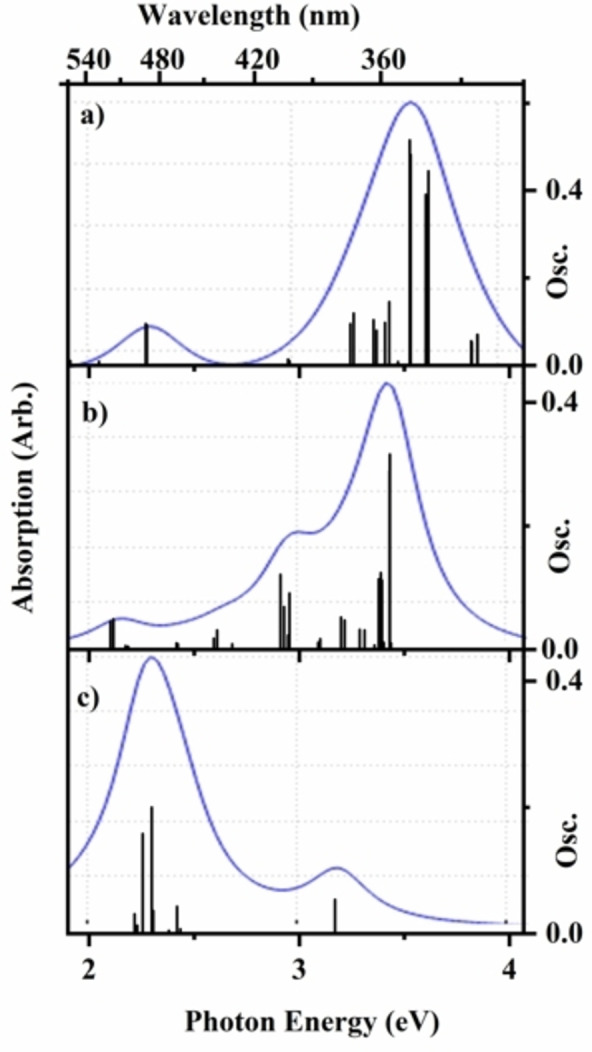
Calculated TD‐DFT (PBE0/6‐31G*) excitation energies (unshifted) for FeTTP^+^ with a) S=1/2 b) S=3/2 and c) S=5/2 Fe spin‐states. Oscillator strengths (Osc.) of individual transitions >0.005 are given by the vertical bars, while the full‐line spectrum is a convolution of the calculated excitations (Lorentzian function, 0.25 eV FWHM).

**Figure 2:**
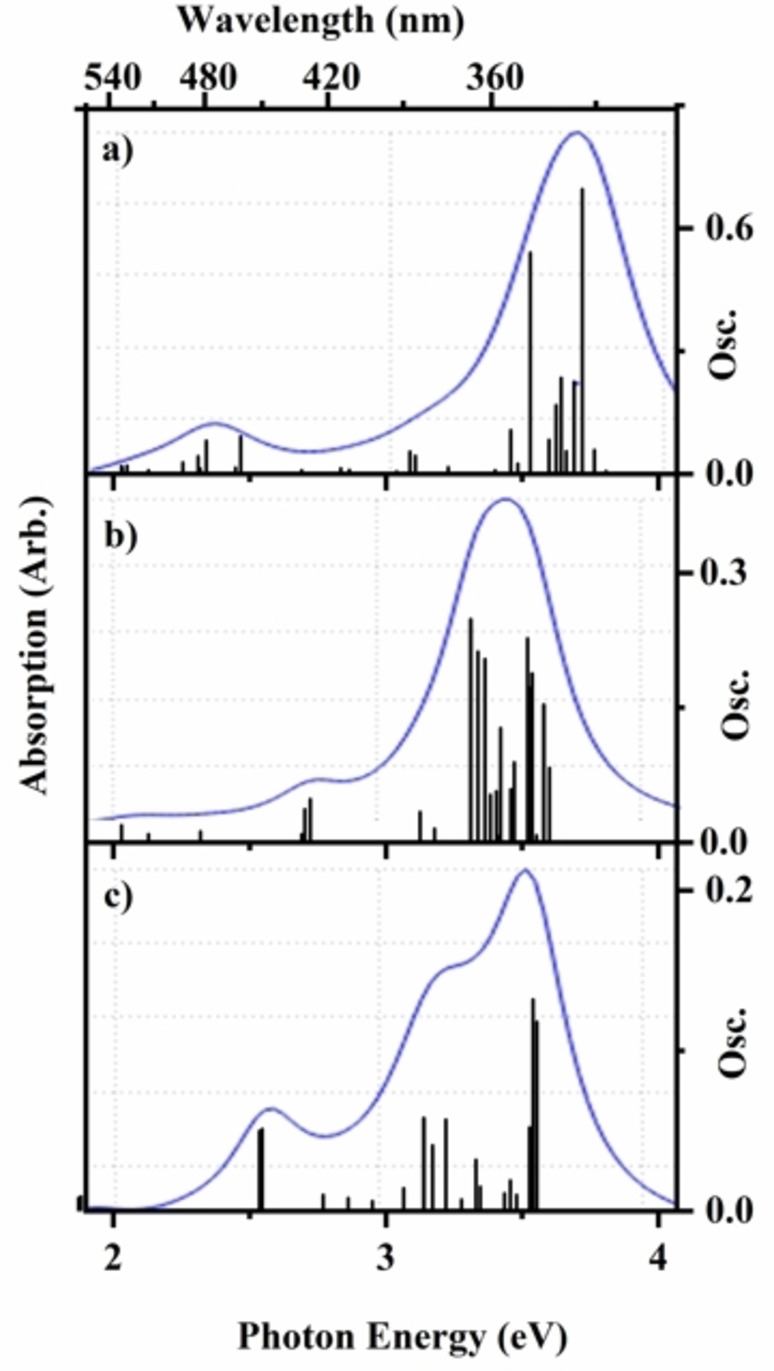
Calculated TD‐DFT (PBE0/6‐31G*) excitation energies (unshifted) for the N‐coordination structures of FeTTP^+^⋅py, with a) S=1/2 b) S=3/2 and c) S=5/2 Fe spin‐states. Oscillator strengths (Osc.) of individual transitions >0.005 are given by the vertical bars, while the full‐line spectrum is a convolution of the calculated excitations (Lorentzian functions).

Figures 1 and 2 present the TDDFT (PBE0/6‐31G*) calculated electronic spectra of FeTPP^+^ and FeTPP^+^⋅py, respectively. The characteristic Q (VIS region) and Soret (UV region) bands are visible in all of the calculated spectra. (The solution phase UV‐VIS spectrum of FeTPPCl is shown in Section S8 for comparison.) While the adduct will possess certain electronic excitations which are localised primarily on the pyridine moiety (i. e. ones associated only with the pyridine chromophore within the adduct), these transitions appear at energies (>4.7 eV) which is beyond the range studied here. Details of the electronic transitions are given in Sections S5 and S6. For both FeTPP^+^ and FeTPP^+^⋅py, the calculations reveal that the bright electronic excitations correspond to π‐π* transitions across both the Q and Soret regions, with electron density variously distributed across the porphyrin and/or phenyl substituents. The most striking aspect of the spectra is the extent to which the Fe spin‐state within the FeTPP^+^ moiety dramatically affects the electronic spectrum, both in terms of band position and the relative intensities of the predicted bands.

### 3.2 Collision‐Induced Dissociation

Low‐energy collision‐induced dissociation (CID) of FeTPP^+^⋅py was conducted to perform an initial characterisation of the adduct obtained via ESI (Section S7). The onset for CID occurs at 5.1 % CID energy, consistent with the adduct being stable prior to collisional excitation.[^56^, ^57^] CID resulted only in simple cluster fission:
(3)

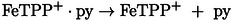




This fragmentation pattern confirms that the initial clusters are composed of intact molecular components of FeTPP^+^ and pyridine monomer units,^[58]^ with no low‐energy reactive fragmentation pathways present.

**Figure 3:**
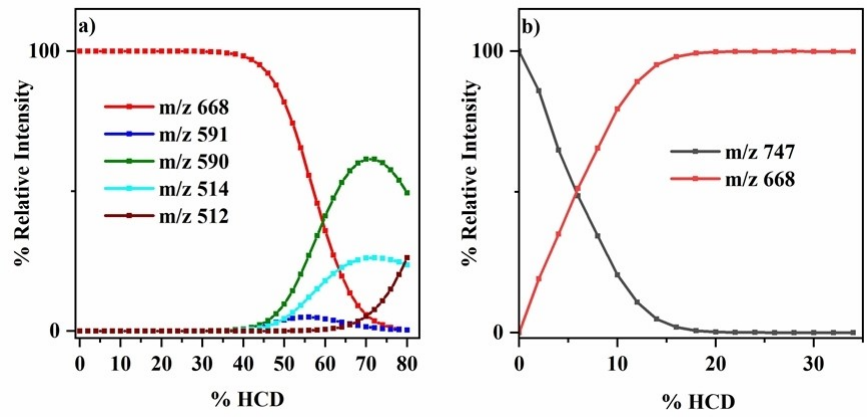
Parent ion dissociation curves of a) FeTPP^+^ [0–80 % HCD energy], and b) FeTPP^+^⋅py [0–35 % HCD energy].

Higher‐energy collisional dissociation (HCD) energy results are presented in Figure 3 for FeTPP^+^ and FeTPP^+^⋅py. For FeTPP^+^, the onset for molecular dissociation occurs around 40 % HCD. This is in contrast to the FeTPP^+^⋅py adduct where cluster fission is observed from around 6 % HCD, reflecting the relatively weaker nature of the intermolecular bond between the MP and pyridine compared to the intramolecular MP bonds. Multiple fragmentation channels are present at higher collision energies for both FeTPP^+^ (4a–4d) and for FeTPP^+^⋅py (5a–5e:
(4a)

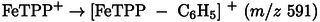



(4b)

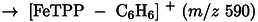



(4c)

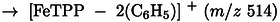



(4d)

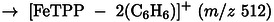



(5a)

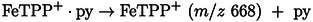



(5b)

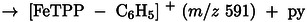



(5c)

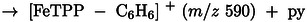



(5d)

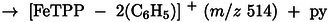



(5e)

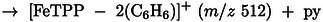




The fragmentation pathways of FeTPP^+^ are all present for the FeTPP^+^⋅py adduct, albeit at an extremely low % dissociation cross section (therefore omitted from Figure 3b), and can be classified as secondary fragments of FeTPP^+^⋅py produced following initial cluster fission [5a]. Bohme and co‐workers studied tetraphenyl iron^III^ and manganese^III^ porphyrin CID previously, and observed similar fragmentation channels associated with loss of phenyl, benzene and bi‐phenyl units.^[59]^

### 3.3 Photodepletion Spectra

#### 3.3.1 Assignment of Spectra

**Figure 4:**
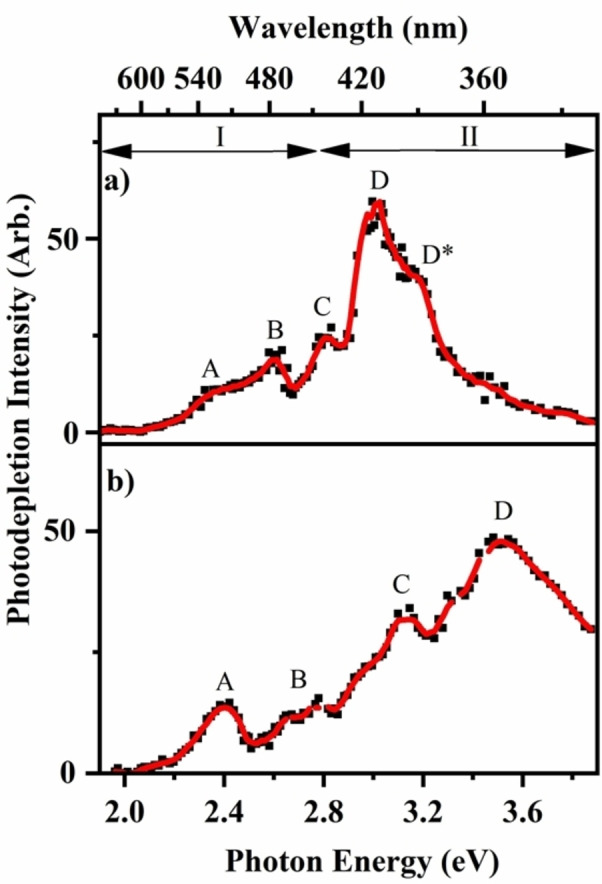
Photodepletion (absorption) spectra of (a) FeTPP^+^ and (b) FeTPP^+^⋅py from 1.9–3.85 eV (652–322 nm). The solid red line is a five‐point adjacent average of the data points.

The effect of complexation on the electronic spectrum of the FeTPP^+^⋅py adduct was explored by acquiring laser photodissociation spectra of FeTPP^+^ and FeTPP^+^⋅py (Figure 4) from 652–302 nm (1.9–4.1 eV). Spectra are acquired via photodepletion of the mass‐selected ion of interest by measuring the reduction in intensity of the precursor ion as a function of laser wavelength. (Photodepletion spectra are equivalent to gas‐phase absorption spectra, subject to a number of conditions.[^60^, ^61^]) Note that luminescene does not need to be considered for iron porphyrins since all transitions are believed to be radiationless.^[62]^

For both systems, photodepletion is observed across the regions labelled I and II which broadly correspond to the MP Q and Soret band regions, respectively. The most intense absorption is observed in the Soret band region for each of the systems, as expected.[^32^, ^63^, ^64^] Spectral features evident on the photodepletion spectra are labelled A–D* for FeTPP^+^ and A–D for FeTPP^+^⋅py, and are discussed further below.

The FeTPP^+^ photodepletion spectrum (Figure 4a) displays absorption in the Q Band region (I) with spectral features (A–C) from 2.40–2.81 eV, as well as an intense peak in the Soret Band region (II) that is centred at 3.15 eV, with a shoulder, labelled (D*), visible to higher energy. This feature can be assigned to a vibronic progression, which is evident in higher‐resolution spectra of porphyrins.[^65^, ^66^] The FeTPP^+^ gas‐phase absorption spectrum displays a very similar band profile as the analogous FeTPPCl solution‐phase absorption spectrum (Section S3). For the gaseous MP, the Soret band peaks at 3.15 eV while the corresponding solution‐phase feature appears at 2.95 eV. Such a gas‐phase to solution‐phase red shift of the Soret band is typical of similar porphyrin systems.[^67^, ^68^]

Figure 4b displays the FeTPP^+^⋅py photodepletion spectrum, which differs from the FeTPP^+^ photodepletion spectrum in several ways. The most intense, Soret band feature (D) of FeTPP^+^ blue‐shifts by 0.47 compared to the comparable peak (D) in the cluster spectrum at 3.52 eV. This result can be compared to that observed by Nielsen and co‐workers from their study of NO complexation to the Fe^III^ heme cations, where a 0.21 eV blue shift of the Soret band occurred.^[32]^ The FeTPP^+^⋅py Soret band is significantly broader than the comparable FeTPP^+^ band, possibly due to the fact that the vibronic progression is less resolved for the adduct. Three additional spectral features are evident at 2.40 eV (A), 2.75 (B), and 3.15 (C). Feature C is particularly prominent and distinctive compared to the FeTPP^+^ spectrum.

Comparing the FeTPP^+^ and FeTPP^+^⋅py photodepletion spectra, it is evident that there are significant differences in the profiles of the two spectra, aside from the general blue‐shift of the most intense band in the adduct. Comparison of the experimental and calculated spectra of FeTPP^+^, shows that there is good agreement between the experimental spectrum (Figure 4a) and the S=3/2 calculated spectrum (Figure 1b), with both displaying the A, B, C and D features with similar intensities and spacing. The calculated spectrum displays electronic excitations at higher energies for comparable bands (+0.32 eV, +0.10 eV, +0.23 eV, and +0.23 eV for A, B, C and D, respectively) than the experimental spectrum, as is usual for TDDFT calculations.[^69^, ^70^] For FeTPP^+^⋅py, although the calculations predict that the S=1/2 spin state should be the minimum energy structure, the experimental spectrum displays the closest agreement to the S=5/2 calculated spectrum. Specifically, the calculated spectrum of the adduct displays electronic excitations at similar energies to the comparable bands of the experimental spectrum (−0.18 eV, −0.01 eV, −0.02 eV, and −0.05 eV for A, B, C and D, respectively). Most notably, the calculated S=5/2 spectrum displays the same double‐peak structure for the Soret band as the experimental spectrum, due to the presence of two predicted clusters of electronic transitions around 3.1 and 3.6 eV.

#### 3.3.2 Comparison of Spectral Intensities for FeTPP^+^ and FeTPP^+^⋅py

**Figure 5:**
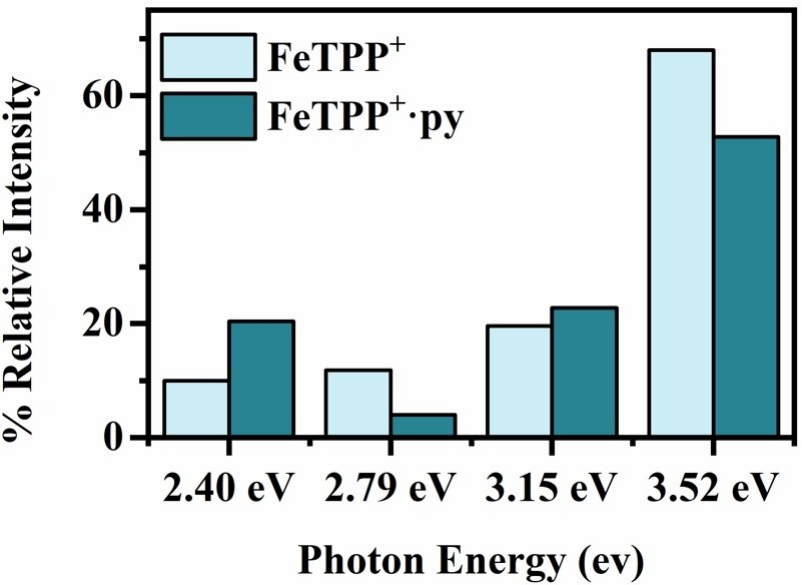
Comparison of the relative % Photodepletion intensities of FeTPP^+^ and Fe.TPP^+^⋅py 2.40 eV, 2.79 eV, 2.90 eV and 3.52 eV photon energies (hv). The photon energies listed are for the FeTPP^+^⋅py adduct: See Section S8 for further details. Experimental errors obtained from repeat runs were ±3 %.

The photodepletion intensities for FeTPP^+^ and FeTPP^+^⋅py displayed in Figure 4 are directly comparable for the two species studied here as the ions are close in *m/*z and therefore have similar detection efficiencies within the MS instrument (i. e. although the intensity is labelled as arbitrary intensity on Figure 4, the photodepletion intensity is directly comparable for the FeTPP^+^ and FeTPP^+^⋅py). Comparison of the photodepletion intensities clearly shows that there is no large decrease in absorption intensity upon binding of pyridine to the MP. Figure 5 presents a more direct comparison for a selection of excitation energies across the Q and Soret bands. (Section S8 gives further details of this comparison.) This shows that there is no dramatic reduction in photodepletion intensity upon pyridine binding. In particular, while photodepletion intensity of FeTPP^+^⋅py is moderately lower than that of FeTPP^+^ in the Soret band region (~3.5 eV), the transition is still very strong. The similar photodepletion intensities of FeTPP^+^ and FeTPP^+^⋅py measured here therefore do not support the theory of Gray and co‐workers’ that MPs lose their signature strong Soret band in UV/VIS spectroscopy upon complexation to aromatic molecules.^[71]^

**Figure 6:**
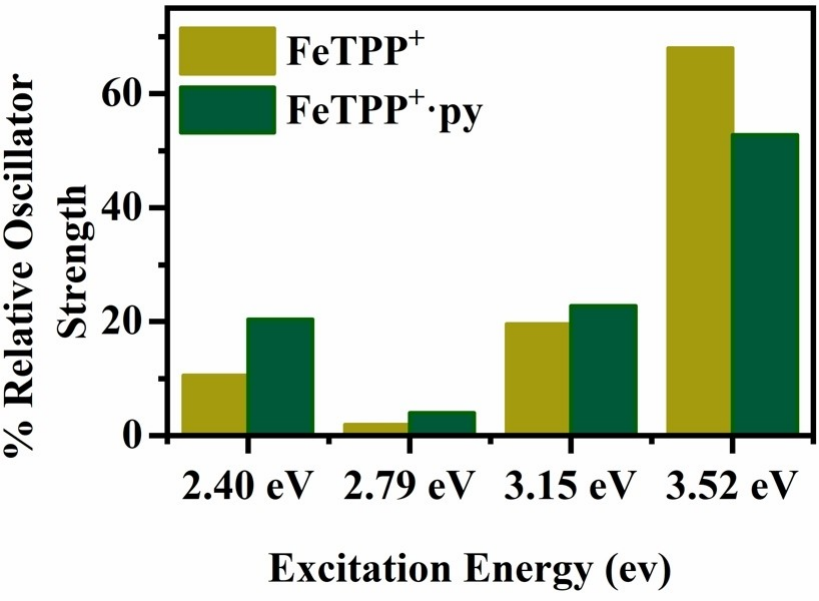
Comparison of the calculated (PBE0/6‐31G(d,p)) % relative oscillator strength for FeTPP^+^ (3/2 spin state) and FeTPP^+^⋅py cluster (5/2 spin state) at 2.40 eV, 2.81 eV, 3.21 eV and 3.52 eV photon energies (hv). See Section S8 for details.

The TDDFT calculations provide further support for the absorption intensity being relatively unaffected on going from FeTPP^+^ to FeTPP^+^⋅py. Figure 6 illustrates the calculated relative absorption, showing that there is no dramatic reduction in absorption on going from FeTPP^+^ to FeTPP^+^⋅py. (See Section S8 for further details.) Interestingly, the relative changes in intensity on going from FeTPP^+^ to FeTPP^+^⋅py at the 2.40, 2.81, 3.15 and 3.52 eV calculated benchmark energies reflect the relative changes seen in the experimental data (Figure 5). For example, the calculations also predict a modest decrease in absorption for the Soret band (3.52 eV) upon complexation.

### 3.4 Photofragmentation of FeTPP^+^ and FeTPP^+^⋅py

**Figure 7:**
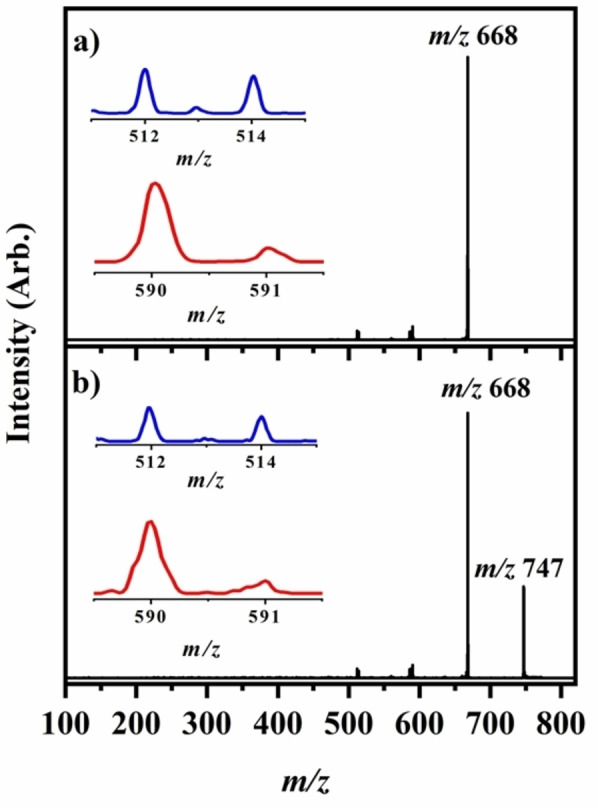
The photofragment mass spectra of a) FeTPP^+^ (*m/z* 668) and b) FeTPP^+^⋅py (*m/z* 747) clusters irradiated at 3.15 eV.

Photodepletion of both FeTPP^+^ and FeTPP^+^⋅py is accompanied by the production of photofragments. Figure 7 displays the photofragment mass spectra of FeTPP^+^ (*m/z* 668) and FeTPP^+^⋅py (*m/z* 747) obtained following excitation at 3.15 eV. FeTPP^+^ photofragments with production of the *m/z* 512, *m/z* 514, *m/z* 590, and *m/z* 591 ions, which mirror fragmentation pathways [4a]–[4d] seen in HCD. Cluster fission dominates the photofragmentation of FeTPP^+^⋅py, with the same *m/z* 512, *m/z* 514, *m/z* 590, and *m/z* 591 ions also produced at very low intensities. No purely photochemical product ions are observed for either FeTPP^+^ or FeTPP^+^⋅py, i. e. all photofragment ions were observed as break down products upon collisional activation.

The observed photofragmentation products all result from rupture of the phenyl substituent‐porphyrin bond, which is notable in the context of the calculated molecular orbitals involved in electronic transitions, i. e. a number of the frontier orbitals appear to involve transfer of orbital density from the phenyl groups onto the porphyrin ring (Sections S4 and S5) consistent with weakening of the phenyl‐porphyrine ring bonds.

A comparison of the relative fragment intensities observed in the HCD and photoexcitation experiments provides insight into the photodynamics of FeTPP^+^ and FeTPP^+^⋅py, as described in full detail in Section S9.[^44^, ^72^, ^73^, ^74^, ^75^]

## 4 Further Discussions

**Figure 8:**
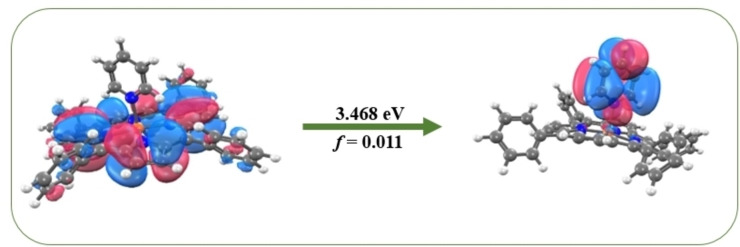
Molecular orbitals involved in the electronic excitation calculated to occur at 3.468 eV for FeTPP^+^⋅py, illustrating the charge‐transfer character of the transition.

Photodissociation spectroscopy of FeTPP^+^ and FeTPP^+^⋅py has shown that pyridine binding to the Fe centered MP can be categorized as a strong binding interaction. This is evident from the switch in the Fe^III^ spin state from S=3/2 to S=5/2 that occurs upon ligand binding as is evident in the dramatic spectral profile change observed upon going from FeTPP^+^ to FeTPP^+^⋅py. The strong binding interaction of pyridine via N‐coordination to the MP seen here mirrors the experimentally determined strong binding energy for imidazole to ferric heme of 213.4 kJ mol^−1^. Interestingly, our calculated binding energy of 116.9 kJ mol^−1^ is closest to the experimental binding energy of the NO ligand to ferri heme (104.1 kJ mol^−1^) amongst the ligands studied by Shafizadeh et al.^[40]^ The strong ligand binding is also evident in our observation of a substantial 0.37 eV (41 nm) blue shift of the Soret band upon adduct formation. This value is comparable to those observed by Nielsen and co‐workers in binding of NO 0.21 eV (23 nm).[^32^, ^76^, ^77^] It should be noted that the substantial blue shift observed here for FeTPP^+^ to FeTPP^+^⋅py incorporates the electronic reorganization of changing from Fe^III^ S=3/2 to S=5/2 that occurs upon ligand binding, so is not a straightforward change in the strength of ligand binding to the MP chromophore between the ground and excited states. However, such blue‐shifts are generally recognized as being indicative of charge‐transfer character electronic transitions. Figure 8 illustrates the TDDFT calculated (Section S6) molecular orbitals involved in brightest electronic transition for FeTPP^+^⋅py in the Soret band region, i. e. at 3.468 eV. A dramatic shift in electron density is evident, from the HOMO‐1 to the LUMO+2, consistent with a charge‐transfer transition.

For both FeTPP^+^ and FeTPP^+^⋅py, the observed photofragments are the same as the observed HCD fragments, although the relative intensities of the various fragments do differ to varying extents at different excitation energies (Section S9). This reveals that non‐statistical photodecay pathways exist for both FeTPP^+^ and FeTPP^+^⋅py. It is possible that these are due to intersystem crossings to triplet states, which introduce bottlenecks for dissociation due to rate‐limiting spin flips being required to re‐reach the electronic ground states.[^32^, ^76^, ^77^] Intriguingly, the data for the FeTPP^+^⋅py adduct is more statistical than for FeTPP^+^ (cross‐comparing fragment intensities) which suggests that the electronic decay pathways are modified on adduct formation, likely due to the different spin states of Fe in FeTPP^+^ and FeTPP^+^⋅py. It is interesting to note that adduct formation, via the induced metal spin flip and associated change in electronic state, appears to enhance the photostability of the MP moiety.

Nonetheless, it is important to acknowledge that multiple photodecay pathways may be present for both FeTPP^+^ and FeTPP^+^⋅py, and the situation across the full spectral range will be complex. Focusing on the FeTPP^+^⋅py cluster as an illustration of this complexity (Section S9), photodecay in the band A region can be characterized as largely statistical, consistent with decay via a conical intersection to a geometry close to that of the electronic ground state. This contrasts with the situation for B and C where the photodissociation is considerably more non‐statistical, showing that longer excited state lifetimes are present in this region.

Finally, the photodepletion results presented here clearly demonstrate that adduct formation with an N‐aromatic does not cause a substantial reduction in the extinction coefficient of the optically bright Soret band of the Fe porphyrin, as was postulated by Stoyanov et al.[^26^, ^27^] Instead, we have found that adduct formation induces a change in the spin‐state of the metal centre of the porphyrin, and leads a substantial blue shift of the Soret band. The origin of the absence of strong UV‐VIS absorptions by MPs in petroleum samples remains an unsolved problem: Possibly the quenching of the electronic transitions is due to porphyrin aggregation as suggested by Dickie et al.^[25]^ The results presented here demonstrate that gas‐phase, mass‐selected spectroscopy experiments provide a technique that would be well suited to testing this hypothesis, via acquiring the spectra of porphyrin dimers and trimers.

## Conflict of Interests

The authors declare no conflicts of interest.

5

## Supporting information

As a service to our authors and readers, this journal provides supporting information supplied by the authors. Such materials are peer reviewed and may be re‐organized for online delivery, but are not copy‐edited or typeset. Technical support issues arising from supporting information (other than missing files) should be addressed to the authors.

Supporting Information

## Data Availability

The data that support the findings of this study are available from the corresponding author upon reasonable request.
