# Supplementary material for: N‐Aromatic Complexation in Tetraphenyl Porphyrin Iron (III)‐Pyridine: Evidence of Spin‐Flip via Gas‐Phase Electronic Spectroscopy
Source: Chemphyschem. 2024 Nov 8;25(24):e202400669. doi: 10.1002/cphc.202400669 (PMC11648826; doi:10.1002/cphc.202400669)
Supplement: Supplementary file 1 — Supporting Information [file CPHC-25-e202400669-s001.pdf]

ChemPhysChem

Supporting Information

N-Aromatic Complexation in Tetraphenyl Porphyrin Iron (III)-Pyridine: Evidence of Spin-Flip via Gas-Phase Electronic Spectroscopy

Kelechi O. Uleanya, Sarah A. Wilson, and Caroline E. H. Dessent*

Supplementary Information

N-Aromatic Complexation in Tetraphenyl Porphyrin Iron (III)-Pyridine: Evidence of Spin-Flip via Gas-Phase Electronic Spectroscopy

Kelechi O. Uleanya, Sarah A. Wilson, and Caroline E. H. Dessent*

Department of Chemistry, University of York, Heslington, York, YO10 5DD, United Kingdom.

ORCID

Kelechi O. Uleanya: 0000-0003-2017-1360

Sarah A. Wilson: 0000-0001-5914-5085

Caroline E. H. Dessent: 0000-0003-4944-0413

S1: Laser energy measurements of the photodepletion of $FeTTP^+$ and $FeTTP^+$ ·py

S2: Geometric structures of $FeTPP^+$ (S = 3/2)

S3: Geometric structures of $FeTPP^+$·py (S = 5/2)

S4: Solution-phase spectrum of FeTPPCl in MeCN

S5: TDDFT calculated molecular orbitals of the $FeTPP^+$ (S = 3/2)

S6: TDDFT calculated molecular orbitals of the $FeTPP^+$·py (S = 5/2)

S7: Collision induced dissociation (CID) of $FeTPP^+$·py

S8: Spectra band shifts for different spectral regions of the $FeTPP^+$ and $FeTPP^+$·py absorption spectra

S9: Discussion on the photodynamics of $FeTPP^+$ and $FeTPP^+$·py from comparison of photofragmentation and HCD fragmentation

S1: Laser energy measurements of the photodepletion FeTTP^{+} and FeTTP^{+} ·py

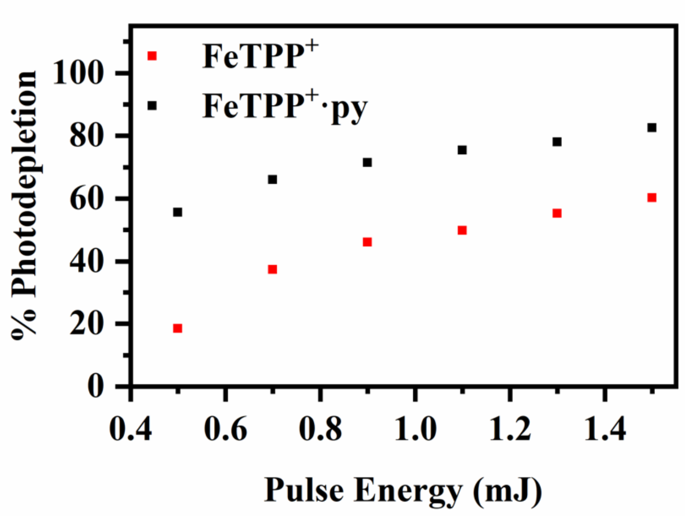

Figure S1: % Photodepletion of the FeTPP^{+} and FeTPP^{+}.py cluster cations measured at 3.02 eV as a function of laser pulse energy (mJ).

S2: Geometric structures of $FeTPP^+$ (S = 3/2)

Table S1: Different orientations of the lowest-energy structure (S=3/2) of $FeTPP^+$

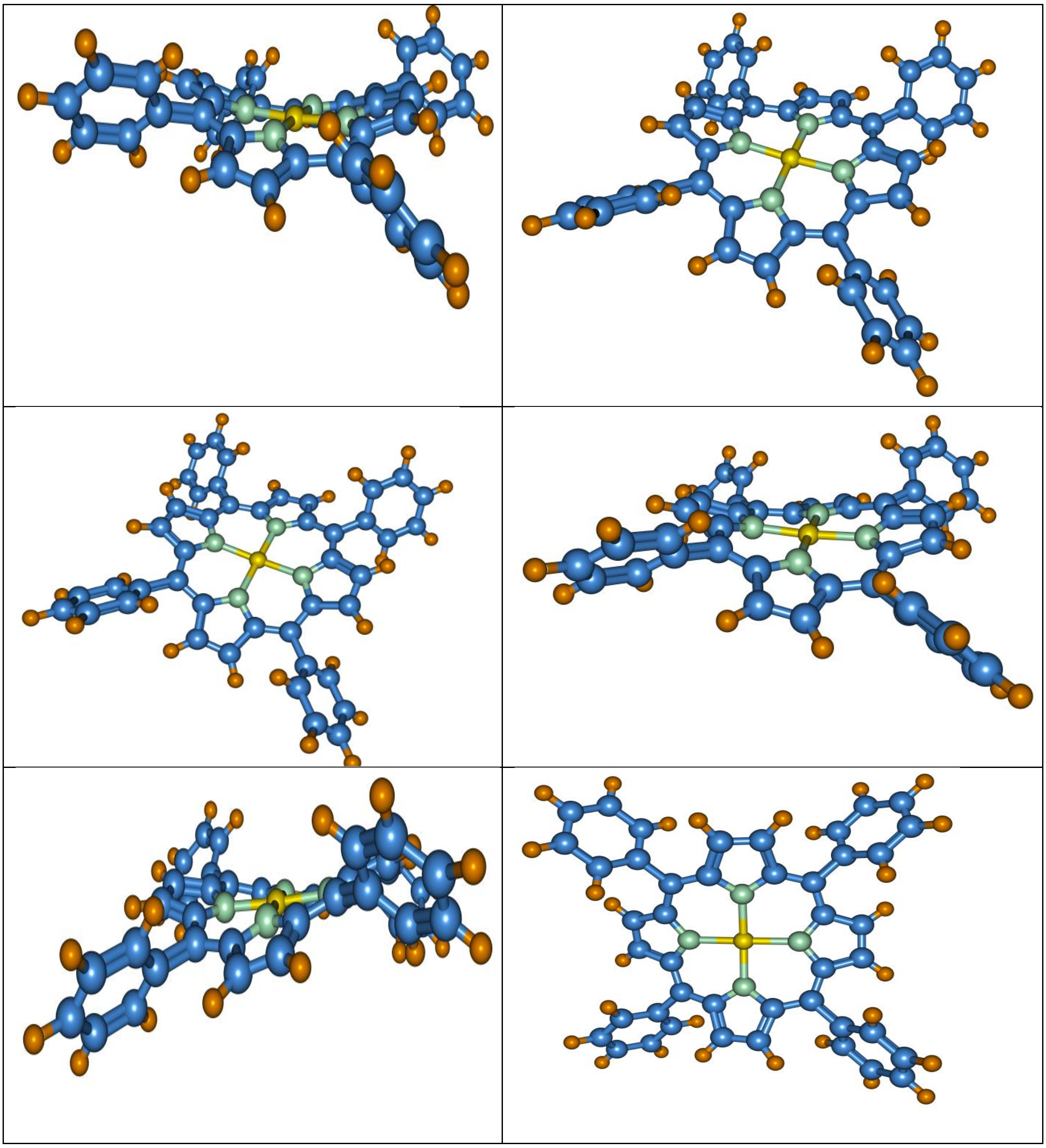

Atoms: C = blue, H = Orange, N = Green, Fe = Yellow

S3: Geometric structures of $FeTPP^{+}\cdot py$ (S = 5/2)

Table S2: Different orientations of the lowest-energy structure (S=5/2) of $FeTPP^{+}\cdot py$

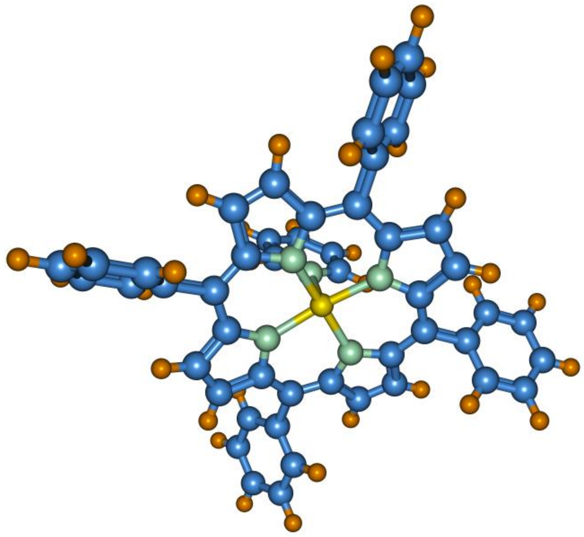	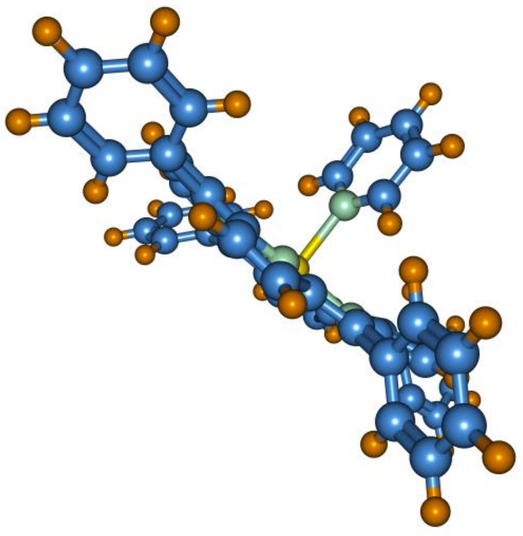
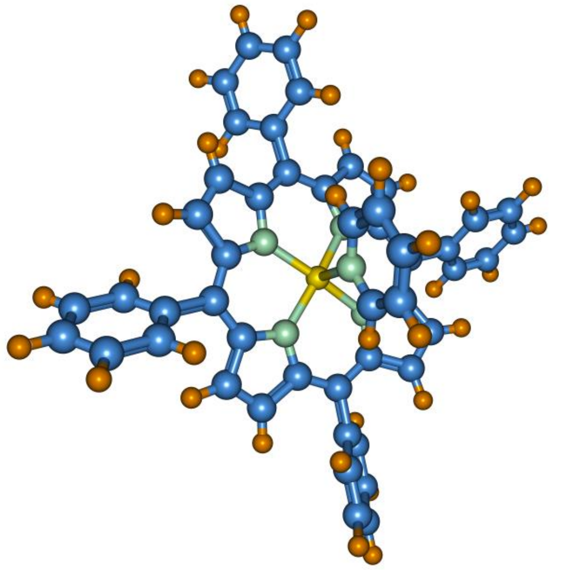	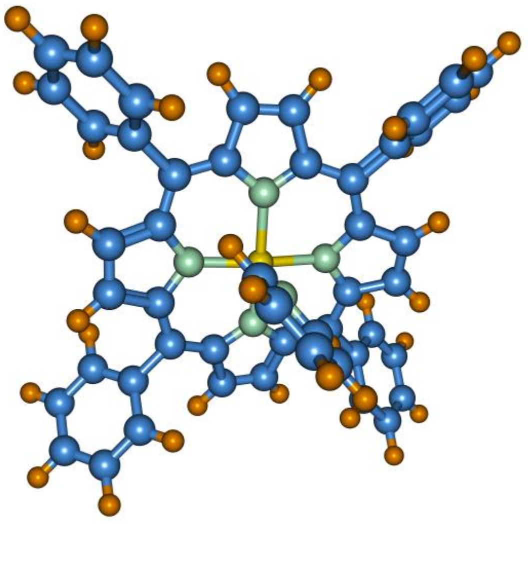
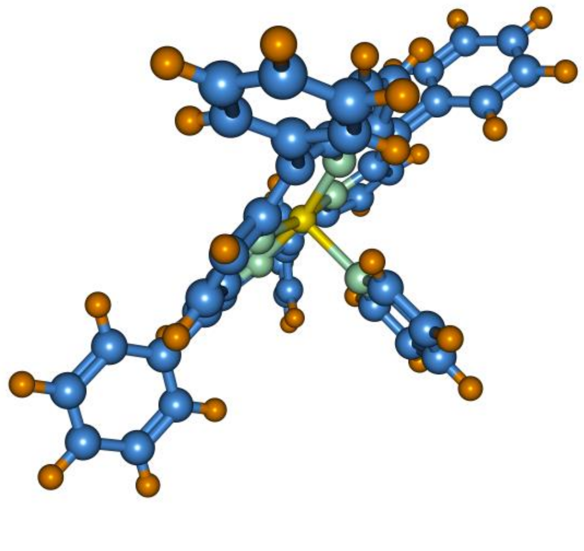	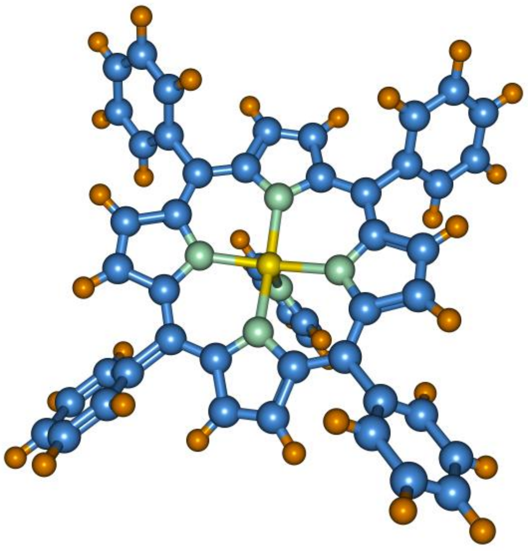

Atoms: C = blue, H = Orange, N = Green, Fe = Yellow

S5: Solution-phase spectrum of FeTPPCl in MeCN

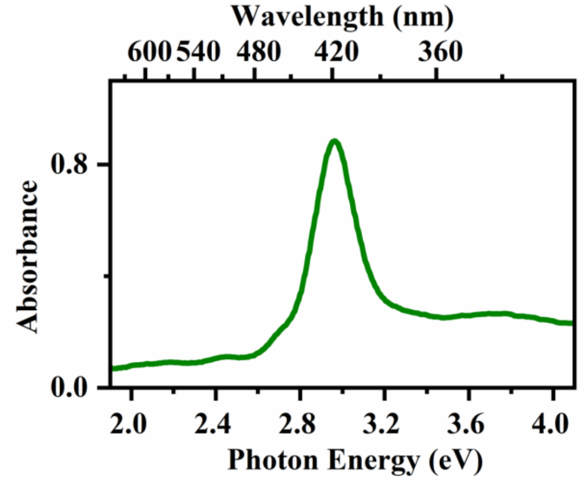

Figure S2: Absorption spectrum of FeTPPCl at (1×10^{-6} mol dm^{-3}) in MeCN.

S5: TDDFT calculated molecular orbitals of the $FeTPP^+$ (S = 3/2)

For $FeTPP^+$ (S = 3/2), the calculated TDDFT electronic excitations correspond to strong π-π* transitions across both the Q and Soret regions. In the Q band region (1.90-2.98 eV), the most intense transitions occur from doubly-occupied molecular orbitals ≤ MO171 to the singly occupied molecular orbitals MO172 and MO174. Although there are some transitions to the LUMO (MO175) and LUMO+1 (MO176) at excitation energies ≤ 2.54 eV, the strongest contributing transitions which are (≥ 0.70) in this region are to the HOMO (MO174) which is a SOMO. The strongest contributing doubly-occupied MO (MO163) corresponds to an orbital which is delocalised across the porphine moiety but with not on the substituent phenyl groups. MO167 and MO168 both display delocalised character on the substituent phenyl groups. In the Soret region (3.00-4.10 eV), the strong contributing transitions at (3.06-3.22 eV) showed similar characteristics to the Q region transitions, i.e. from doubly occupied MO161 and MO162 orbitals to the HOMO and HOMO-1. The most intense Soret region transitions (3.26-3.50 eV) occur to the LUMO or LUMO +1. It is notable that the HOMO (MO174) corresponds to an orbital which is delocalised across the entire $FeTPP^+$ moiety except for the metal, while the LUMO (MO175) is delocalised on the porphyrin scaffold and metal, but not on the phenyl substituent groups.

Table S3: Calculated TDDFT transition energies at the PBE0/6-31G(d) level of theory and oscillator strengths of $FeTPP^+$ (S = 3/2). MO transitions with oscillator strength > 0.005 are represented below.

Orbital transitions	ΔE (eV)	f
174 (π) (HOMO) → 177(π*)(LUMO + 2)<br>171 (π) → 175(π*) (LUMO)<br>171(π) → 176(π*)(LUMO +1)	2.149	0.049
163(π) → 175(π*)(LUMO)<br>164(π) → 175(π*)(LUMO)	2.207	0.006
163(π) → 175(π*)(LUMO)<br>164(π) → 175(π*)(LUMO)	2.219	0.005
172(π) → 175(π*)(LUMO)	2.449	0.010
171(π) → 175(π*)(LUMO)	2.456	0.009

173(π) → 176(π*)(LUMO +1)<br>171(π) → 175(π*)(LUMO)	2.626	0.018
173(π) → 176(π*)(LUMO +1)<br>173(π) → 177(π*)<br>170(π) → 175(π*)(LUMO)<br>171(π) → 176(π*)(LUMO +1)	2.642	0.031
169(π) → 172(π*)<br>164(π) → 172(π*)<br>165(π) → 172(π*)	2.714	0.009
160(π) → 172(π*)<br>170(π) → 175(π*)(LUMO)<br>171(π) → 176(π*)(LUMO +1)	2.944	0.121
161(π) → 174(π*)(HOMO)<br>166(π) → 174(π*)(HOMO	2.961	0.069
160(π) → 172(π*)<br>168(π) → 175(π*)(LUMO)<br>160(π) → 174(π*)(HOMO)	2.979	0.023
160(π) → 172(π*)<br>170(π) → 176(π*)(LUMO + 1)	2.987	0.091
162 (π) → 174(π*)(HOMO)	3.122	0.011
160(π) → 173(π*)(HOMO-1)	3.133	0.017
167(π) → 175(π*)(LUMO)<br>168(π) → 175(π*)(LUMO)<br>160(π) → 174(π*)(HOMO)	3.232	0.052
168(π) → 175(π*)(LUMO)	3.249	0.047
160(π) → 174(π*)(HOMO)	3.321	0.032
161(π) → 174(π*)(HOMO)<br>163(π) → 174(π*)(HOMO)<br>166(π) → 174(π*)(HOMO)	3.343	0.031
162(π) → 173(π*))<br>170(π) → 176(π*)(LUMO + 1)	3.411	0.114
162(π) → 173(π*)<br>162(π) → 174(π*)(HOMO)	3.420	0.124

167(π) → 177(π*)<br>162(π) → 174(π*)(HOMO)	3.426	0.112
171(π) → 177(π*)<br>172(π) → 176(π*)(LUMO+1)	3.435	0.011
169(π) → 177(π*)<br>170(π) → 177(π*)<br>171(π) → 175(π*)(LUMO)	3.462	0.289
169(π) → 177(π*))<br>170(π) → 175(π*)(LUMO)<br>170(π) → 176(π*)(LUMO+1)	3.464	0.316

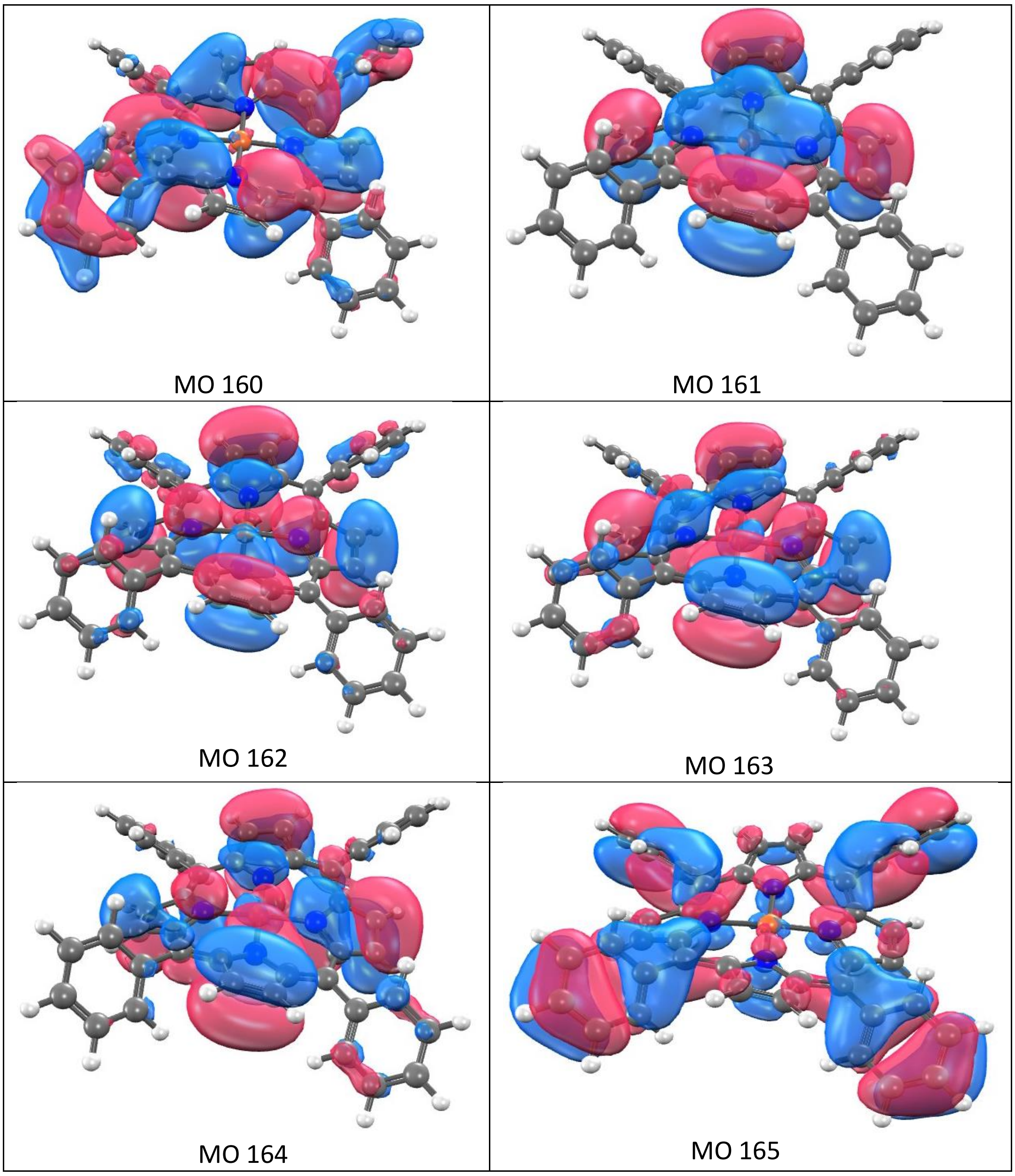

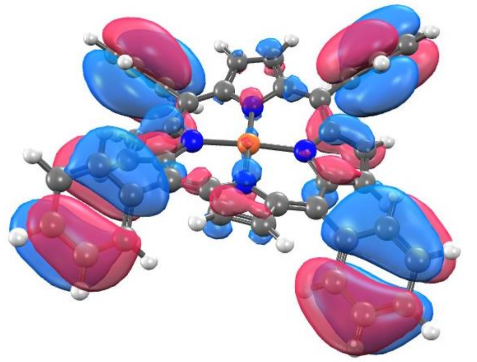

MO 166

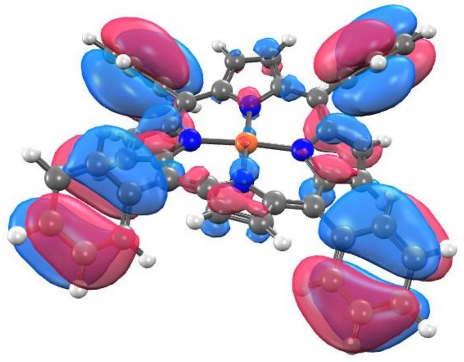

MO 167

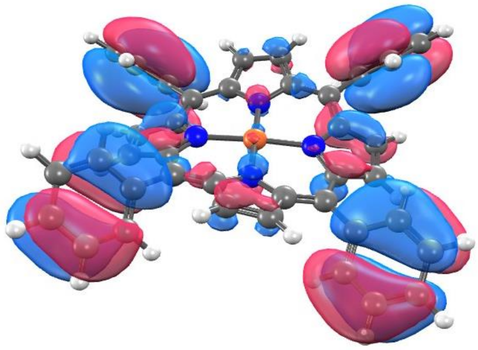

MO 168

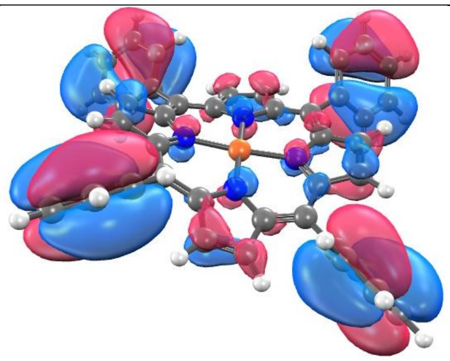

MO 169

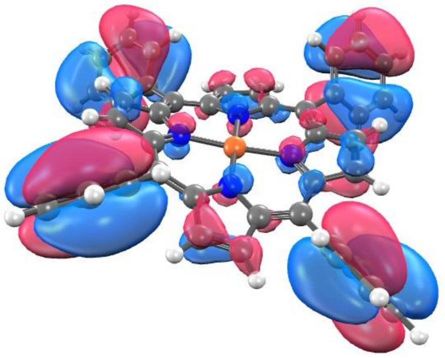

MO 170

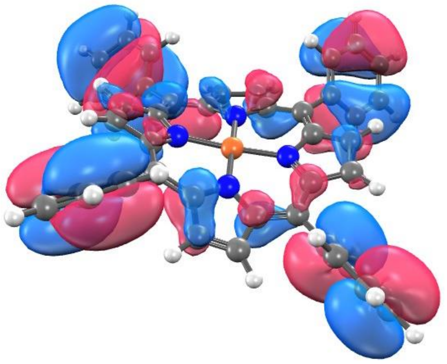

MO 171

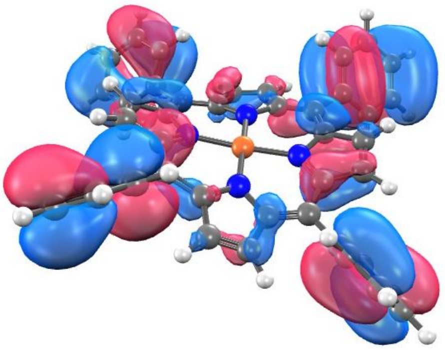

MO 172 (SOMO)

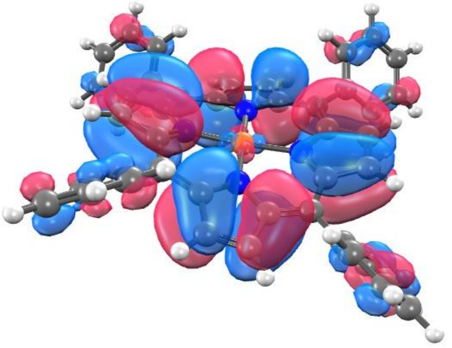

MO 173(HOMO - 1) (SOMO)

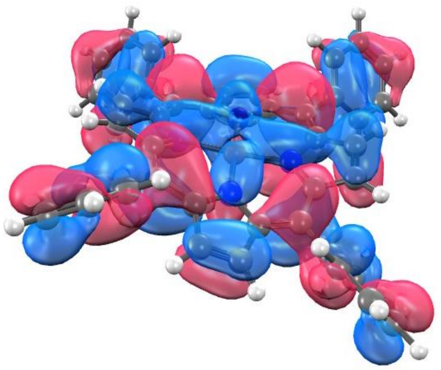

MO 174(HOMO) (SOMO)

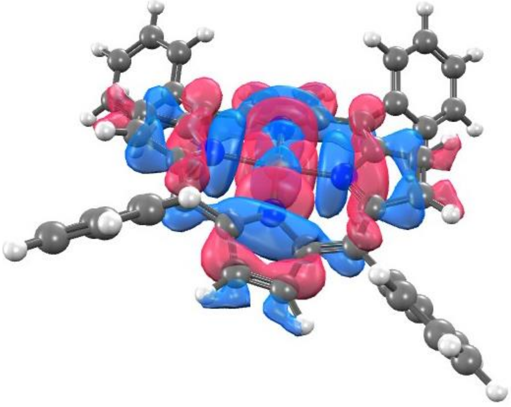

MO 175(LUMO)

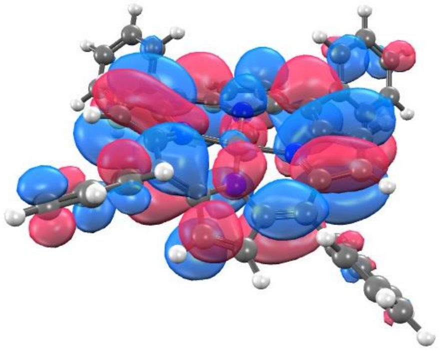

MO 176(LUMO + 1)

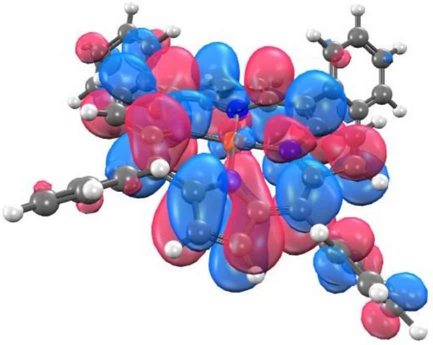

MO 177(LUMO + 2)

S6: TDDFT calculated molecular orbitals of $FeTPP^{+}\cdot py$ (S = 5/2)

The TDDFT calculations predict that $FeTTP^{+}\cdot py$ (S = 5/2) displays a smaller number of intense electronic transitions compared to $FeTPP^{+}$ due to the different spin states of the iron. Unlike $FeTPP^{+}$, the majority of the contributing transitions in the Q region (1.90 – 2.98 eV) are transitions from the doubly occupied MOs (≤ MO191) to the LUMO or LUMO+1. In the Soret region (3.00 – 4.10 eV) of $FeTTP^{+}\cdot py$, although there are some transitions to the LUMO and LUMO+1 from doubly-occupied MOs (178, 180, 182, 187, 189), the major contributing transitions are from the doubly occupied MO to singly occupied MO (192,193,) or HOMO-1 (MO 195). This is in contrast to the observed transitions in $FeTPP^{+}$ in this region.

Notably, the brightest transition of $FeTTP^{+}\cdot py$ is predicted to be a charge-transfer transition. The HOMO orbital (MO196) displays a delocalised orbital that encompasses the whole porphyrin moiety including the metal centre while the LUMO (MO197) constitutes an orbital that is delocalised around the porphine scaffold but not the metal or phenyl groups.

Table S4 Calculated TDDFT transition energies at the PBE0/6-31G(d) level of theory and oscillator strengths of $FeTPP^{+}\cdot py$ (S = 5/2). MO transitions that contributed more than 20% to the excitations and with oscillator strength > 0.005**.**

**Orbital transitions**	**ΔE (eV)**	**f**
196 (π) (HOMO) → 197(π*)(LUMO)<br>190 (π) → 196(π*)(HOMO)<br>191(π) → 197(π*)(LUMO)	2.569	0.050
196(π) (HOMO) → 198(π*)(LUMO +1)<br>190(π) → 197(π*)(LUMO)<br>191(π) → 196(π*)(HOMO)	2.580	0.051
187(π) → 192(π*)	2.803	0.010
187(π) → 193(π*)	2.894	0.008
181(π) → 192(π*)<br>183(π) → 192(π*)	2.983	0.006
181(π) → 193(π*)<br>183(π) → 193(π*)	3.098	0.014
178(π) → 192(π*)	3.172	0.058

182(π) ⟶ 192(π*)		
190(π)⟶ 197(π*)(LUMO)<br>191(π) ⟶ 197(π*)(LUMO)	3.204	0.041
178(π)⟶ 193(π*)<br>182(π) ⟶ 193(π*)<br>178(π) ⟶194(π*)<br>191(π) ⟶197(π*)(LUMO)	3.253	0.057
178(π) ⟶ 195(π*)(HOMO - 1)<br>182(π) ⟶194(π*)	3.310	0.007
178(π) ⟶194(π*)<br>180(π) ⟶195(π*)(HOMO -1)<br>184(π) ⟶194(π*)<br>189(π)⟶195(π*)(HOMO -1)	3.363	0.032
188(π) ⟶ 195(π*)(HOMO - 1)<br>189(π)⟶195(π*)(HOMO -1)	3.379	0.014
195(π) (HOMO -1)⟶ 199(π*) (proposed CT)	3.468	0.011
180(π) ⟶ 192(π*)<br>192(π) ⟶198(π*)(π*)(LUMO + 1)<br>189(π) ⟶ 195(π*)(HOMO - 1)	3.489	0.019
189(π) ⟶ 195(π*)(HOMO - 1)<br>192(π)⟶ 197(π*)(LUMO)	3.513	0.010
183 (π)⟶197(π*)(LUMO)<br>190(π)⟶ 197(π*)(LUMO)<br>192(π)⟶ 198(π*)(π*)(LUMO + 1)	3.560	0.052
192(π) ⟶197(π*)(LUMO)<br>192(π)⟶ 198(π*)(π*)(LUMO + 1)	3.573	0.132
192(π) ⟶ 197(π*)(LUMO)<br>192(π)⟶ 198(π*)(π*)(LUMO + 1)<br>190(π) ⟶ 197(π*)(LUMO)	3.587	0.118

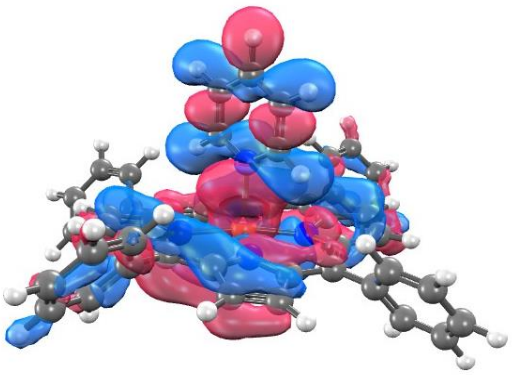

MO 178

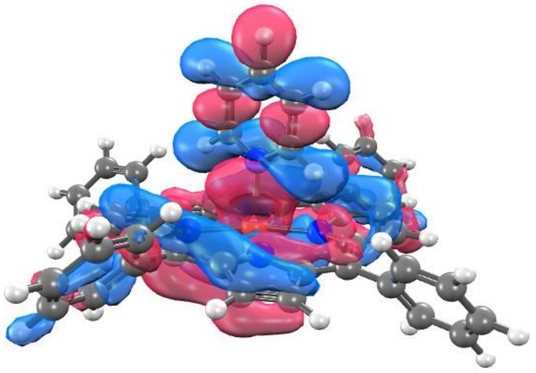

MO 180

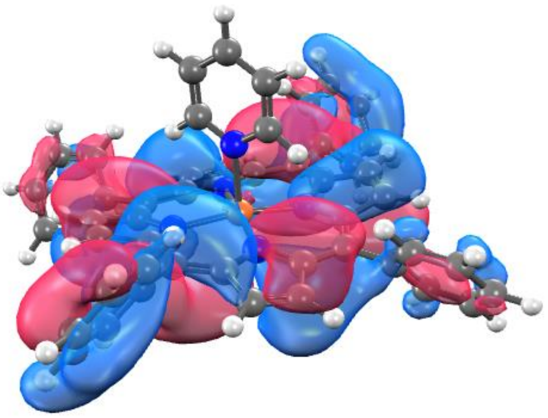

MO 181

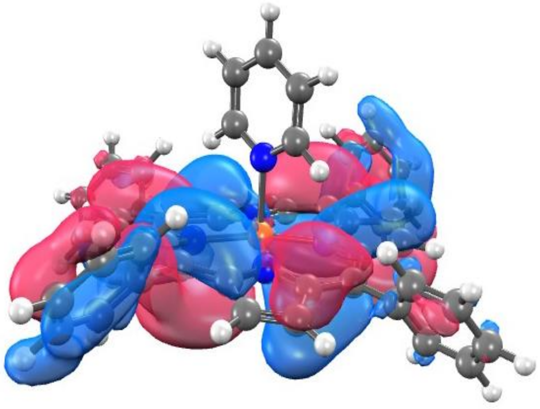

MO 182

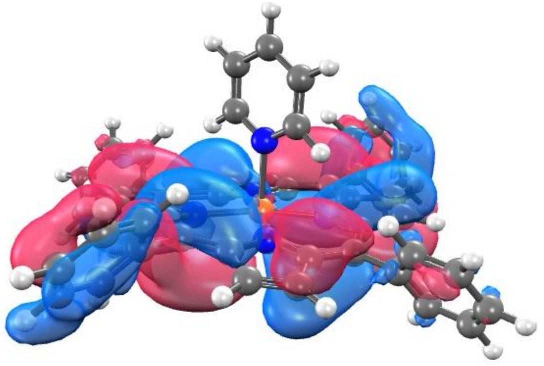

MO 183

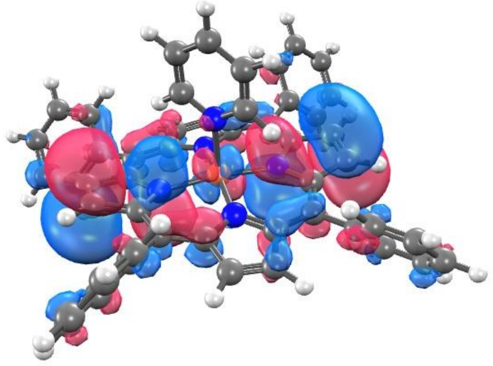

MO 184

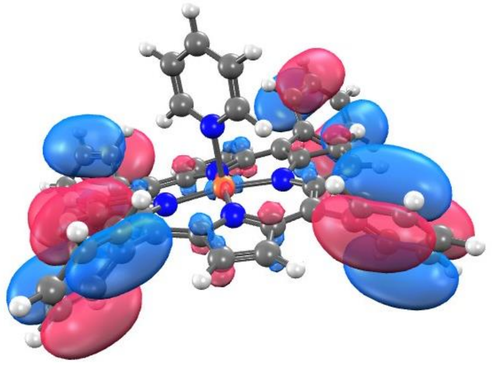

MO 187

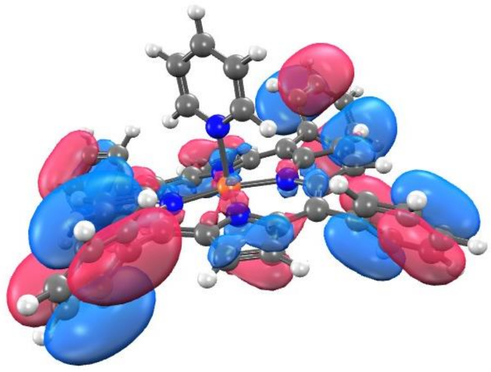

MO 189

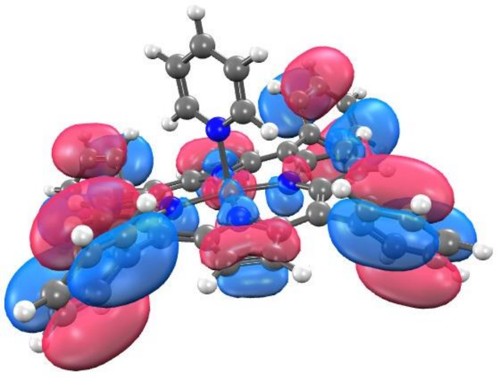<br>MO 190	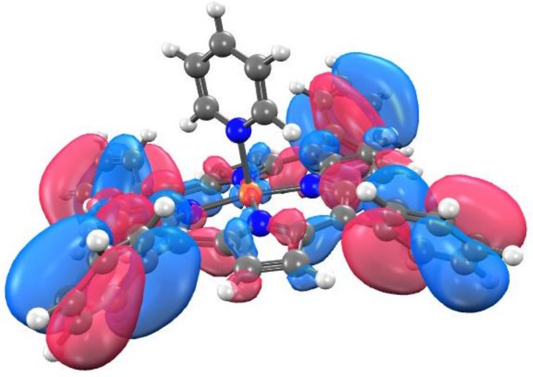<br>MO 191
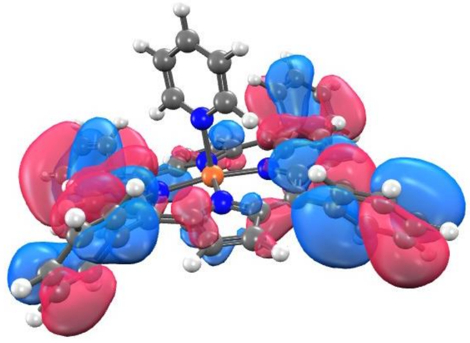<br>MO 192 (SOMO)	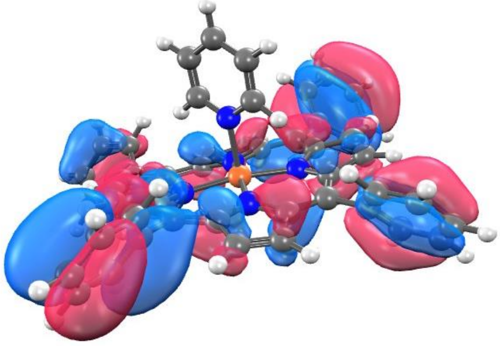<br>MO 193 (SOMO)
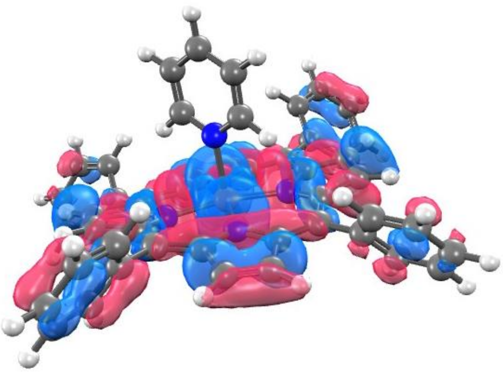<br>MO 194 (SOMO)	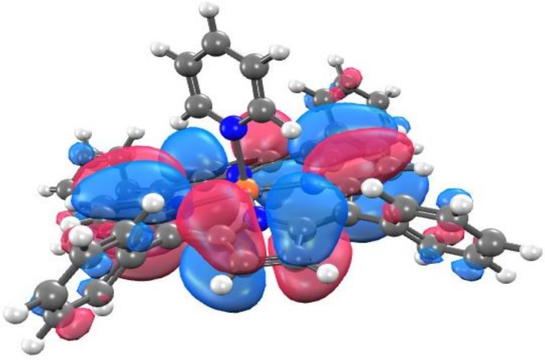<br>MO 195 (HOMO - 1) (S0MO)
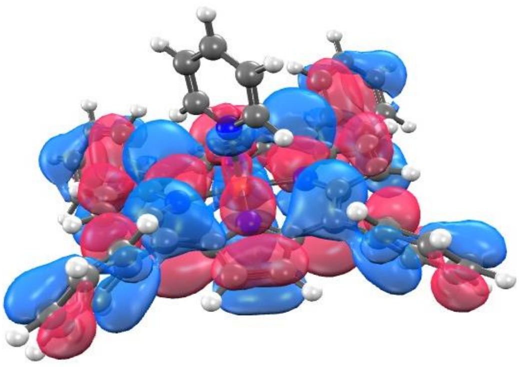<br>MO 196 (HOMO) (SOMO)	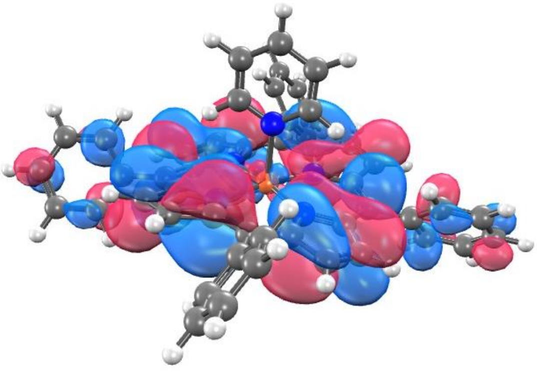<br>MO 197 (LUMO)

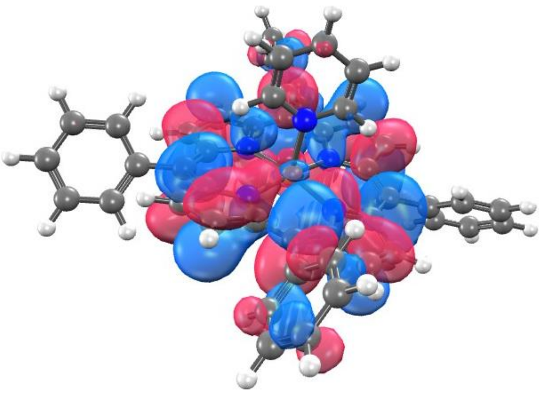<br>MO 198 (LUMO + 1)	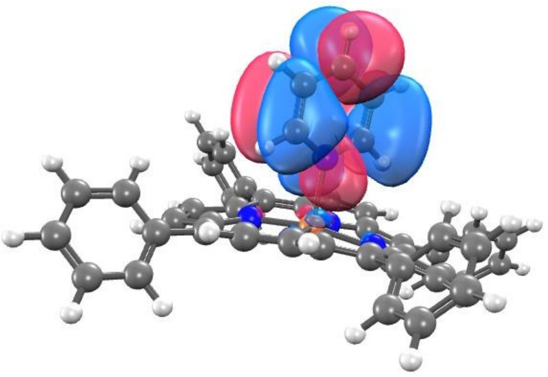<br>MO 199

S7: Collision induced dissociation (CID) of $FeTPP^{+}$·py

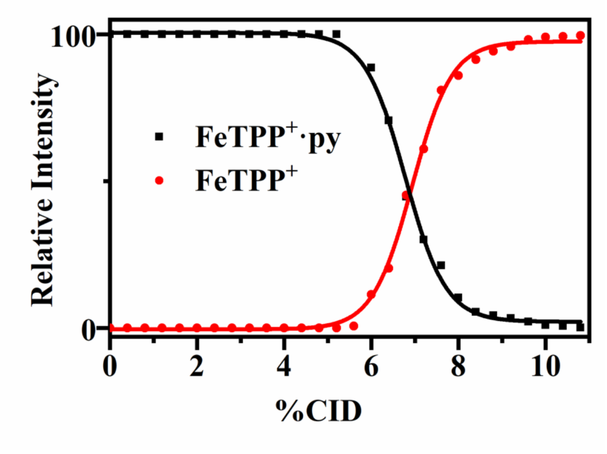

Figure S3: Parent ion dissociation curves for $FeTPP^{+}$·py alongside fragment production curves upon low-energy CID between 0 and 10% CID energy. Standard experimental errors obtained from repeat runs were ± 3%.

S8: Spectra band shifts at different spectral regions of the $FeTPP^+$ and $FeTPP^+$·py absorption spectra

To obtain a meaningful comparison of relative spectral intensity, differential spectral shifts were applied to provide the best like-for-like comparison between $FeTPP^+$ and $FeTPP^+$·py. (It was not possible to compare the photodepletion intensities at the same photon energies for $FeTPP^+$ and $FeTPP^+$·py due to the spectral shift that occurs upon complexation.) To complicate this situation, it is also not possible to apply a single value of spectral shift across one adjusted spectrum, since the spectral bands (A-D) shift by different extents. We have therefore applied a specific spectral shift at each of the selected photon energies so that the intensities are compared for equivalent spectral features.

Table S5: $FeTPP^+$ band shifts for the different spectral regions of the photodepletion spectra, compared to the corresponding $FeTPP^+$·py absorption bands.

**Photon energy (hv)**	**2.40 eV**	**2.79 eV**	**3.15 eV**	**3.52 eV**
**$FeTPP^+$**	-0.06	0.00	+0.25	+0.37
**Laser energy (mJ)**	0.5	0.5	0.3	0.1

The different energies represents the absorption features A-D in Figure 4 of the main manuscript.

As with the experimental data, that calculated TDDFT absorption intensities are also compared at adjusted excitation energies to allow a comparison of the calculated intensities of similar spectral features. (This comparison does need to be treated with caution since calculated TDDFT intensities for electronic transitions are known to be unreliable.)

Table S6: TDDFT Band shifts at the different regions of the compared experimental electronic absorption bands.

**Photon energy (hv)**	**2.40 eV**	**2.79 eV**	**3.15 eV**	**3.52 eV**
**$FeTPP^+$**	+0.26	+0.08	+0.21	+0.06

**$FeTPP^+$·py**	-0.18	-0.01	-0.02	-0.05

The different energies represents the absorption features A-D in Figure 4 of the main manuscript.

For the monomer, 2.46 eV, 2.79 eV, 2.90 eV and 3.15 eV correspond to 2.14 eV, 2.71 eV, 2.94 eV, and 3.46 eV energies of the transitions respectively while for the pyridine adduct, 2.40 eV, 2.79 eV, 3.15 eV and 3.52 eV correspond to 2.58 eV, 2.80 eV, 3.17 eV, and 3.57 eV energies

S9: Discussion on the photodynamics of $FeTPP^{+}$ and $FeTPP^{+}\cdot py$ from comparison of photofragmentation and HCD fragmentation

A detailed comparison of the relative fragment intensities observed in the HCD and photoexcitation experiments provides insight into the photodynamics through an assessment of whether photodissociation can be characterised as statistical or non-statistical at various excitation energies. [44,72-75] In "non-statistical decay", the photofragments obtained will be different (i.e. in terms of their identities and relative intensities) from the ground electronic state fragments seen following HCD. Non-statistical decay dynamics are associated with dissociative breakdown pathways occurring directly from the excited state without the involvement of a conical intersection that could return the system to a geometry that is similar to the electronic starting geometry. (A further discussion on identifying statistical versus non-statistical photodissociation can be found in Ref. 70 of the main manuscript).

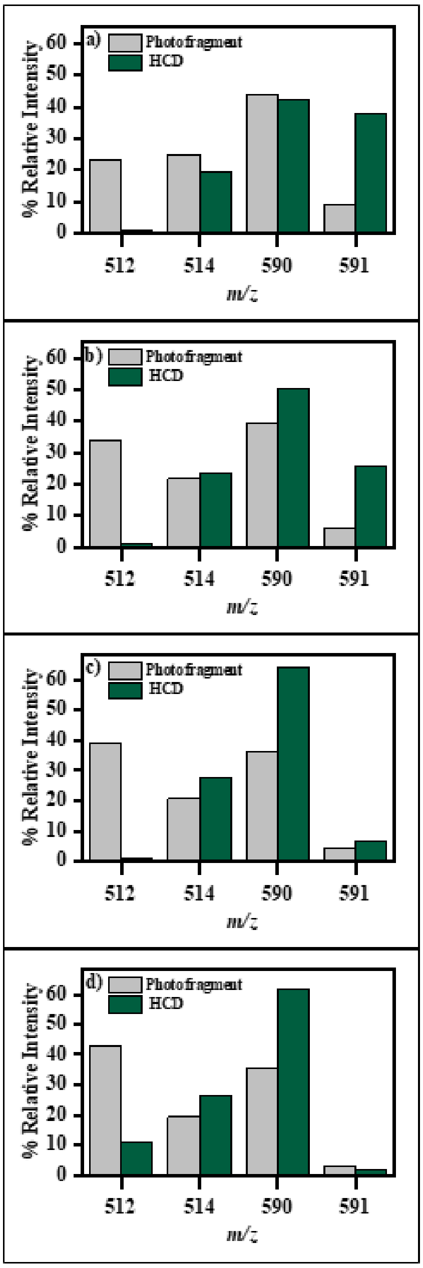

Figure S4: Comparison of the % Photofragment and % HCD fragment yield for $FeTPP^+$ at a) 2.46 eV / 40%, b) 2.79 eV / 48%, c) 2.90 eV / 60% and d) 3.15 eV /74% respectively. Photofragment % relative intensities are from the photodepletion spectra band maxima.

Figure S4 presents a comparison of the % Photofragment and % HCD fragment yield for $FeTPP^+$. For statistical dissociation to be present, the relative fragment yields should be similar

for all of the fragment ions at a given excitation energy. From the intensity comparison presented in Figure S4, it is clear that the relative intensities of fragment production are not equivalent for HCD and photoexcitation at any of the key photon energies. This reveals that non-statistical photodissociation pathways are active. In particular, it is notable that photochemical production of *m/z* 512 is enhanced at all photoexcitation energies.

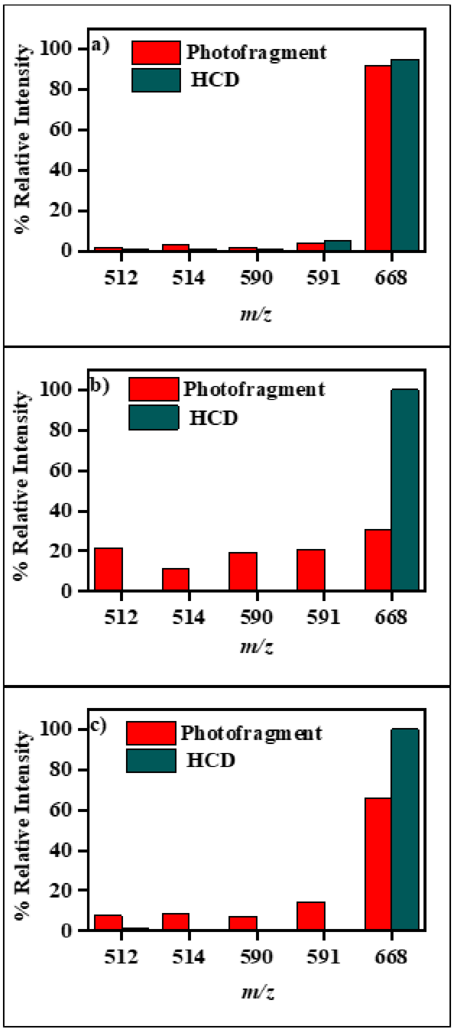

Figure S5: Comparison of the % Photofragment and % HCD fragment yield for $FeTPP^{+}$·py at a) 2.40 eV / 0.2 %, b) 3.15 eV / 6.1 % and c) 3.52 eV / 16 % respectively. Note: the photofragments are the % relative intensities at the actual photodepletion spectrum band maxima.

As noted above, photofragmentation of $FeTPP^+ \cdot py$ is dominated by cluster fission with production of $FeTPP^+$ as the primary photofragment. Figure S5 provides some further detail of how the relative intensities of the full set of photofragments for $FeTPP^+ \cdot py$ vary at different photon energies. For photoexciation at 2.40 eV (Band A: Figure S5a), the HCD and photofragments intensities are reasonably similar, indicating that statistical fragmentation occurs at this excitation energy. The situation is distinctly different at 3.15 eV (Band C: Figure S5b), since the relative intensity of the *m/z* 512, *m/z* 514, *m/z* 590, and *m/z* 591 is now enhanced compared to their HCD energies, indicating that non-statistical dissociation is present. The relative intensity profiles at 3.52 eV (Band D: Figure S5c) lie somewhere intermediate between those at 2.40 eV and 3.15 eV, indicating that some non-statistical dissociation is again evident.
